# Concentration-Dependent Control of the Band Gap Energy of a Low-Dimensional Lepidocrocite Titanate

**DOI:** 10.1021/acsnano.4c16410

**Published:** 2025-01-16

**Authors:** Adam D. Walter, Gregory R. Schwenk, Yuanren Liu, David Bugallo Ferron, Jeffrey T. Wilk, Lucas M. Ferrer, Christopher Y. Li, Yong-Jie Hu, Michel W. Barsoum

**Affiliations:** Department of Materials Science and Engineering, Drexel University, Philadelphia, Pennsylvania 19104, United States

**Keywords:** low-dimensional, lepidocrocite, titanate, quantum confinement, band gap energy, liquid crystal

## Abstract

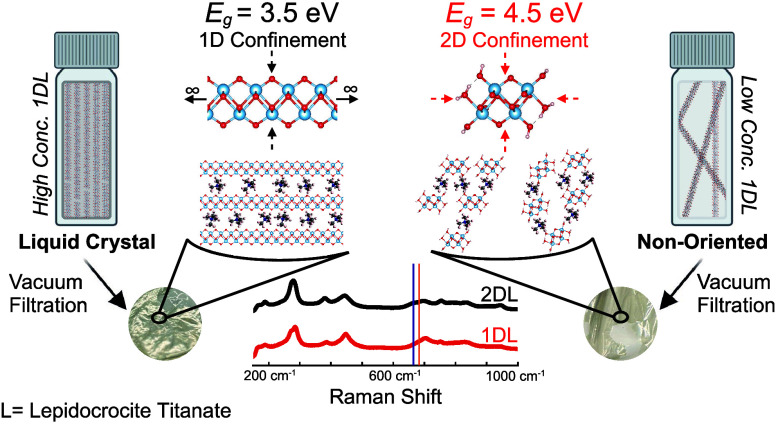

Recently, we reported on the simple, scalable synthesis of quantum-confined one-dimensional (1D) lepidocrocite titanate nanofilaments (1DLs). Herein, we show, using solid-state UV–vis spectroscopy, that reducing the concentration of aqueous 1DL colloidal suspensions from 40 to 0.01 g/L increases the band gap energy and light absorption onset of dried filtered films from ≈3.5 to ≈4.5 eV. This range is ascribed to quantum confinement as the system transitions from two-dimensional (2D) into 1D with dilution. It is only after the colloidal suspensions are dried and the 1DLs start to self-assemble into ribbons and sheets that the band gap values change. This self-assembly is manifested in the X-ray diffraction patterns and the emergence of a Raman band characteristic of 2D lepidocrocite titanates. In colloidal form, 1DLs exhibit a lyotropic liquid crystal phase with a critical concentration of between 10 and 1 g/L. Additionally, the Beer–Lambert law applies with a mass absorbance coefficient of 2 ± 0.4 Lg^–1^ cm^–1^. The optical absorbance edges of the colloidal suspensions are not a function of concentration. The experimental findings are theoretically supported by density functional theory calculations of the Raman vibrational modes and electronic band structures of the 1D and 2D lepidocrocite titanate atomic structures.

Research relating to the synthesis and application of various semiconducting nanomaterials (SNs)—be they three-dimensional (3D), two-dimensional (2D), or one-dimensional (1D)—has garnered significant interest in both academic and industrial settings.^1−6^ With decreasing sizes, the specific surface areas increase, and eventually, quantum confinement effects are observed, resulting in unique properties that their bulk counterparts lack. Owing to these properties, SNs show remarkable results in areas such as catalysis,^7−9^ drug delivery,^10−13^ air/water purification,^14−16^ and many others.^17−19^ The benefits of an enhanced specific surface area are intuitive (a greater number of potential reactive sites for a given material). Additionally, the discrete energy levels of sufficiently small SNs allow for well-defined transitions, a powerful tool for catalysis and optoelectronic devices so long as the band gap energy (*E*_g_) of the SN fits the desired application.^5,6,20^

There are several established methods to change the *E*_g_ values of SNs. For example, doping can alter *E*_g_ to a degree but introduces additional processing steps and, importantly, changes the chemistry of the system.^21−23^ In some SNs, like phosphorene—the phosphorus analogue of graphene—*E*_g_ may be altered by increasing or decreasing the number of layers;^24,25^ however, this presents drawbacks such as passivation effects that compromise stability and activity.^26−29^ Another method, more germane to this study, is to manipulate the *E*_g_ of a SN by changing its morphology, specifically dimensionality. According to solid-state theory, reductions in dimensionality from 3D to 2D, or from 2D to 1D, result in quantum confinement, which is manifested as an increase in *E*_g_. As such, a great deal of effort has been expended in reducing the dimensionality of SNs in order to manipulate *E*_g_ without compromising their underlying chemical identity.^5,30−32^ For example, in the titanate literature, *E*_g_ increases from the range of ≈3.2 to 3.4 eV^33,34^ to about 3.7 to 3.8 eV as the system goes from 3D to 2D.^35,36^ The 2D nanosheets were obtained by acid treating, and delaminating bulk layered Cs-titanates.^37^ This increase in *E*_g_ is essential in supporting the idea that the delamination was successful, resulting in a 2D structure.

Before proceeding further, it is worth noting that Sasaki et al.^34^ and many others^35,38−42^ assumed lepidocrocite titanate (LT) structures to be indirect semiconductors. However, further development in first-principles density functional theory (DFT) calculations predicts that the 2D LTs are direct band gap semiconductors.^43−45^ Needless to add, whether a material is assumed to be direct or indirect can have important ramifications on its *E*_g_ value, as discussed below.

Recently, 1D LT nanofilaments (1DLs) have been synthesized. This process is mild, simple, and scalable; commercially available titanium precursors are reacted with tetramethylammonium hydroxide (TMAOH)^46,47^ at ambient atmosphere and temperatures <100° for tens of hours in polyethylene bottles. It is believed that the TMA^+^ cation acts as a templating agent upon which the formation of 1DLs depends. As such, when alkali hydroxides are used, instead of TMAOH, alkali titanates are obtained,^48^ similar to those reported in the literature.^49,50^ The simplicity of this fabrication method cannot be overstated, by which an extremely stable 1DL colloidal suspension is obtainable. 1DLs have exhibited cation-exchangeability,^51^ providing exceptional adsorption capacity for actinides^52^ and dyes^53,54^ for water treatment, and even sulfur for use in Li–S batteries.^55^

As indicated by several characterization techniques, 1DLs present a truly 1D building block unit with a minimal cross section of 2 × 2 (height × width) TiO_6_ octahedra and lengths on the order of 10s of nanometers (Figure 1).^46,47,56^ The thicknesses of the 1DL base units are determined to be ≈ 5 Å^47,51,57^ and are consistent with the 2D nanosheets in the literature.^58^ In regard to their widths, the situation is more complex and depends, as shown in this paper, on the concentration of the colloids from which the solid 1DL product is dried. 1DLs can be considered analogous to snippets of 2D LTs. The latter can be considered to be much wider units of 2xZ octahedral units.^34^ In some cases, these sheets get so wide (Z tends to infinity) that they begin to scroll into nanotubes.^50,59,60^

**Figure 1:**
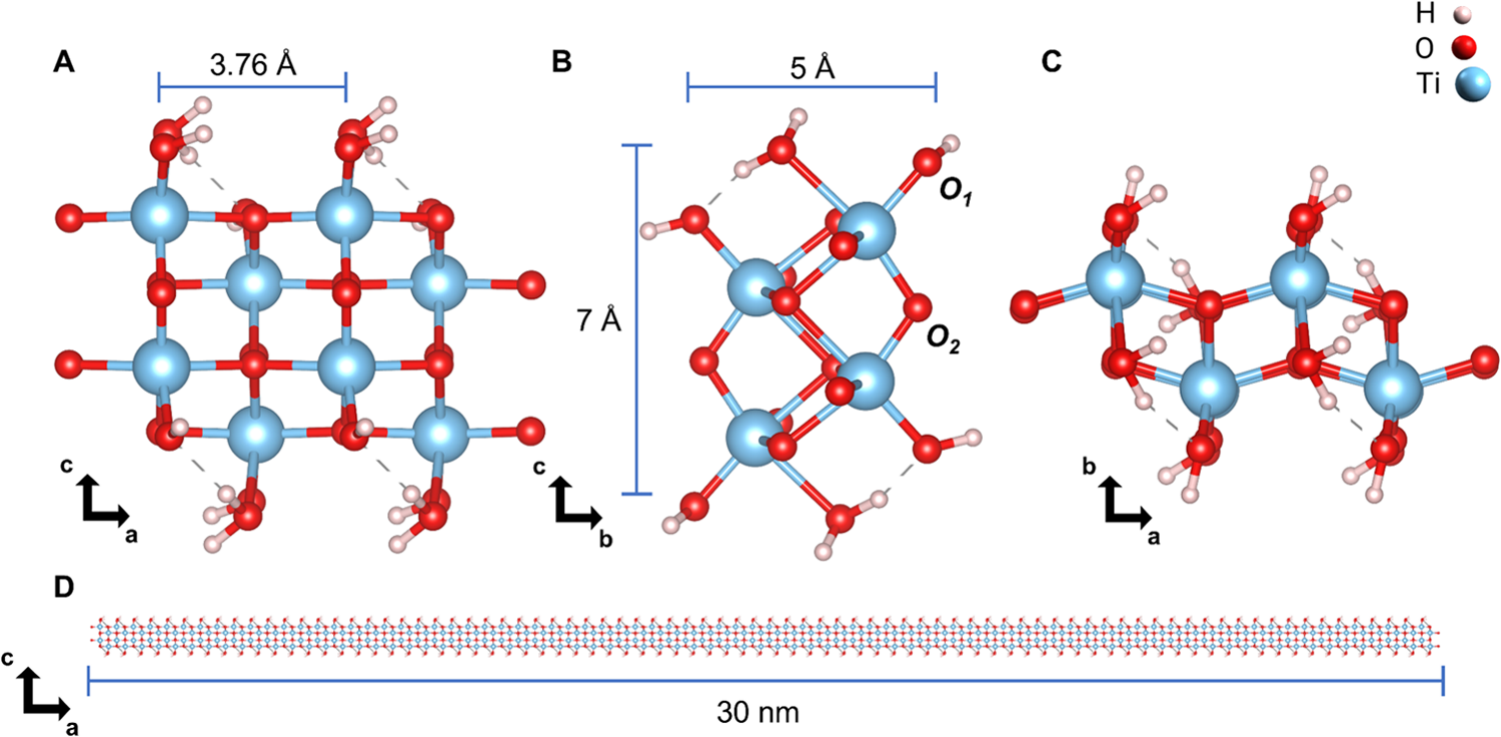
1DL structural base unit. DFT-suggested atomic structure of the 1DL base unit showing the (A) a–c plane, (B) b–c plane; c, (C) a–b plane, and (D) expanded a–c plane—drawn to scale—to emphasize the length and aspect ratio of a typical 1DL Ti–O chain or snippet.

Until recently, there was no direct evidence for the average width of 1DLs in their dry state; two recent papers have shed light on the matter. In the first, cross sections of 1DL filtered films were imaged by scanning transmission electron microscopy (TEM), and filaments with widths in the 1 to 6 nm range (average of 3 nm) were directly observed.^57^ In a second paper, 1DLs were coated with branched polyethylenimine, a cationic polymer, in order to isolate individual filaments.^61^ As the solvent was removed, the mixture self-assembled into hybrid columnar hexagonal phases. According to structural estimations based on X-ray diffraction (XRD), the smallest filament diameter was estimated to be ∼0.6 nm. These results allow for the current understanding that 1DLs exist over a range of widths.

While 1DLs have demonstrated photocatalytic processes like the self-sensitized degradation of wastewater dyes^53,54^ and water splitting,^62^ there has been limited investigation into their electronic properties.^63^ Throughout much of the early work, 1DLs exhibited *E*_g_ values of ≈4 eV, measured by multiple methods.^47,63^ This *E*_g_ is the highest reported in the literature for a titanium oxide-based material and is fundamental to the claim that 1DLs are truly 1D. It is important to note that there is a published body of work on what is traditionally considered “1D” titania.^64−66^ Importantly, to the best of the authors’ knowledge, none exhibit quantum confinement in the form of an enhanced *E*_g_ and are thus not strictly 1D in the quantum mechanical sense.

During more recent experimentation, it became apparent that 1DLs exhibit a wide range of *E*_g_ in their solid-state diffuse reflectance spectra based on the sample preparation method. The purpose of this study was to understand this behavior to control *E*_g_. The shift in *E*_g_ is accompanied by a modification in the structure of the dried 1DL product, which is most easily observable in the XRD patterns and Raman spectra. DFT calculations were performed for the 1D and 2D LT atomic structures to investigate the effects of confinement on electronic band structures and predict Raman spectra to compare with experimental findings.

## Results and Discussion

### 1DL Base Unit

This study focuses on 1DL aqueous colloidal suspensions (Figures S1 and S2), obtainable by adding water to the ethanol-washed product (see the Methods section). Notably, the washed product must remain wet with ethanol before introducing the water. Porous mesostructured particles of agglomerated 1DLs, which do not suspend as a colloid, are obtained if the ethanol-washed product is fully dried prior to the addition of water.^51^

A DFT-suggested unit cell of a single 1DL filament is shown in Figure 1A–C. The 1D periodicity is held along the *a*-direction of the typical 2D LT structure, with an *a*-lattice parameter of 3.76 Å—which corresponds to the periodic translation distance between adjacent Ti atoms along the filament growth direction (Figure 1A). 1DLs generally exist as snippets ≈30 nm in length;^56^ this extreme dimensionality is best seen when a single Ti–O chain is drawn to scale in Figure 1D. As noted above, the minimal cross section of an individual 1DL is simply 2 × 2 TiO_6_ octahedral units or 5 × 7 Å^2^^46,47,56^ (Figure 1B), with a width along the *c*-direction of about ∼6.0 Å, corresponding to the edge-to-edge distance of Ti atoms (Figure 1A). In order to cap the dangling bonds at the edge of the 1DL structure, water molecules or their dissociated –H and/or –OH groups were added. The most stable capping configuration was determined via convex hull analysis of the formation energy from pristine TiO_2_ and water molecules, giving an overall chemistry of (TiO_2_)_1/2_(H_2_O)_1/2_. More details of the calculation results will be reported in a follow-up paper separately.

In reality, along the *b*-, or stacking direction, the distance depends on the nature of the cations in between the 1DLs, as well as the nature of the stacking.^57^ 1DLs exhibit ABA stacking when TMA^+^ cations exist in the interfilamentous space, most likely coordinated at Ti–O–Ti or bridging-O (denoted by O_2_ in Figure 1B), consistent with LT literature. Herein, the *b*-parameter is ≈23.0 Å, which, assuming ABA stacking, corresponds to an interfilamentous distance of 11.5 Å, fitting well to the sum of the diameter of a hydrated TMA^+^^67^ and the height of an individual 1DL filament, ≈5 Å (Figure 1C).

The 1DLs used in this study were prepared as aqueous suspensions; therefore, their propensity to take up protons cannot be dismissed. These may coordinate to both the Ti–O^–^ or terminal oxygen, denoted by O_1_ in Figure 1B, and/or O_2_. Since TMA^+^ is much larger than H^+^, it determines the *b*-parameter. Figure S3 shows two stacked 1DL filaments with TMA^+^ between the filaments, exhibiting ABA stacking. Understanding the complex interaction of water and hydronium with 1DLs is the subject of ongoing work.

### Concentration-Dependent *E*_g_

**Figure 2:**
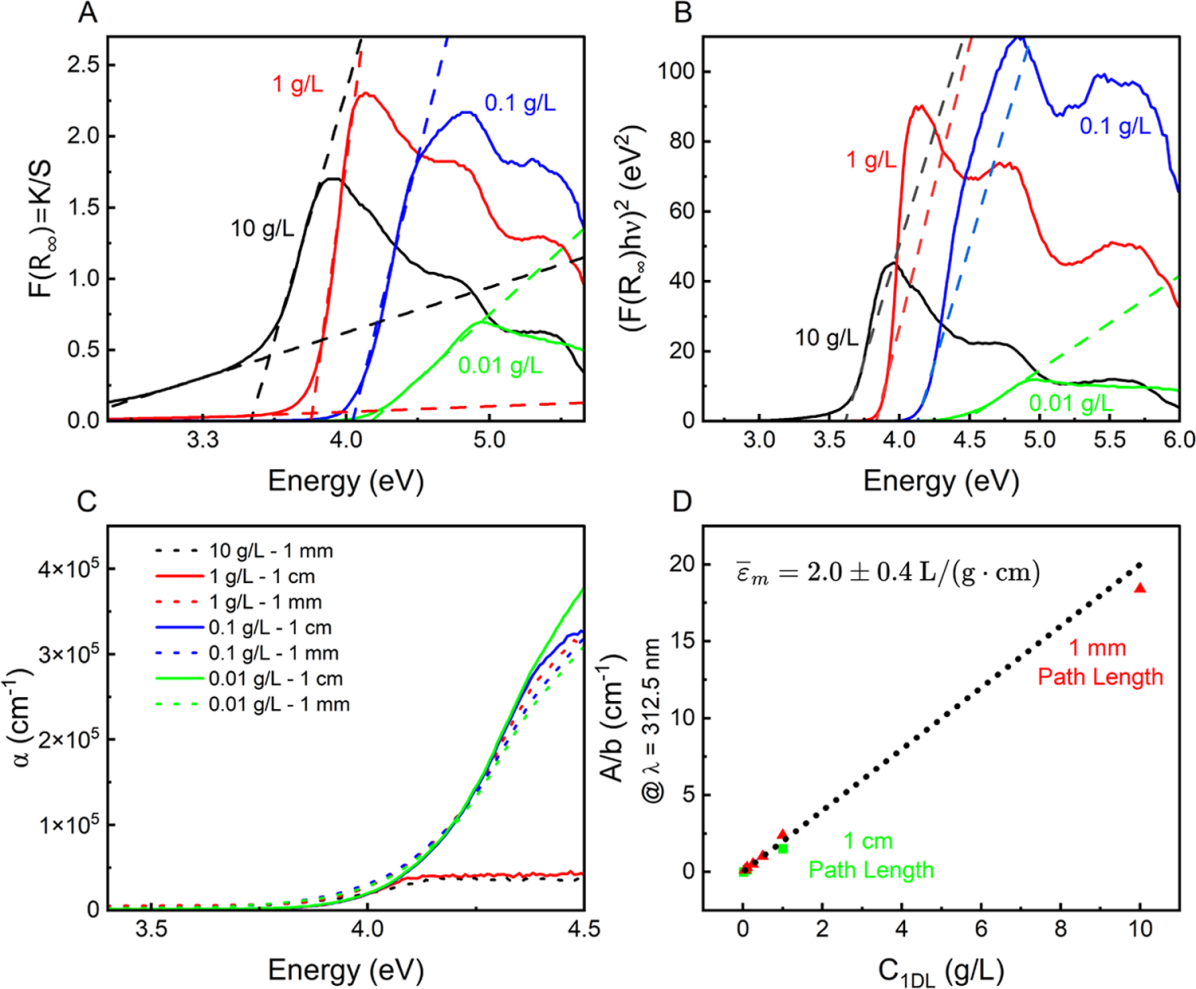
Concentration-dependent optical response of 1DLs. (A) Typical KM plots of 1DL films fabricated by vacuum filtering colloidal suspensions of concentrations indicated on the plot, calculated from UV–vis absorbance spectra obtained by diffuse reflectance (Figure S4). (B) Direct Tauc treatment of (A), as outlined by Makuła et al.^69^ Labels and graphs are color coordinated in panels (A) and (B). (C) α (zero absorption coefficeint) plots of 1DL colloids measured in transmission, treated by the method in Serpone et al.^73^ Raw absorbance is shown in Figure S7, and α over a larger range of values is shown in Figure S8. (D) Absorbance response of 1DL colloids normalized by path length. A linear fit is used to obtain ε̅_m_ for the 1DL colloid.

Figure 2A plots typical solid-state reflectance results (Figure S4) treated with the Kubelka–Munk (KM) function.^68^ The KM function can be described as
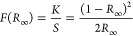
where *R*_∞_ is the reflectance of an infinitely thick sample , *K* is the absorption coefficient, and *S* is the scattering coefficient. Figure 2B shows the former converted into Tauc plots by the method outlined by Makuła et al.^69^ This transformation is outlined by
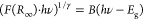
where *h*ν is the energy of the incident photon,*B* is a constant, and γ is the electron transition factor, which depends on whether a semiconductor is direct or indirect, assigned the value of 1/2 or 2, respectively. Here, we assume the 1DLs are direct *E*_g_ semiconductors (see below), but to be consistent with the LT literature,^34,35^ the indirect treatment is available in Figure S4. To ensure this behavior was not an artifact of a single batch of 1DLs, two replicate 1DL batches were prepared, and their optical properties are shown in Figures S5 and S6. The presence of the oscillations at the lower wavelengths shown in Figures S4–S6 may be indicative of quantum sublevel transitions by electrons in the conduction band—seen in quantum dots.^70,71^ It is hereby acknowledged that the Tauc method does not give the best values of *E*_g_ for quantum confined materials, as discussed by Klein et al.^72^ It is useful here, however, since it can be used to compare absorption onset values with previous literature.

As illustrated in Figure 2A,B, the more concentrated the filtered colloid, the lower the absorption onset of the KM treated data and direct *E*_g_, respectively. This narrowing can be attributed to a structure that is less quantum confined at high concentrations. To explain this observation, we hypothesize that, during drying, –OH terminations on two adjacent 1DLs undergo oxolation to form Ti–O–Ti bonds and release water. This is apparently discouraged by increasing the degree of hydration around each 1DL, chiefly by diluting the colloid. Said otherwise, the more hydrated each 1DL unit, the less it can interact with another.

To support the conjecture that drying forces self-assembly, the optical properties of 1DL colloids were studied under various concentration and path length combinations (Figure S7). The colloidal state absorbance response can be converted to α, or zero absorption coefficient, as a function of incident photon energy if one assumes that^73^
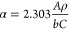
where *A* is the sample absorbance, ρ is the 1DL density (assumed to be 4000 g/L, based on the denisty of anatase and rutile titania), *b* is path length (in cm), and *C* is the sample concentration (in g/L). The plot of α is shown in Figure 2C, showing that the absorption onsets for all colloidal samples, regardless of concentration and path length, are all 4.0 eV. The magnitude of α here is not important, since concentrated samples will absorb nearly all of the light at high energies (Figure S7). In the Tauc method, α is treated as equivalent to *F*(*R*_∞_); thus, comparing Figure 2A–C, it is apparent that drying the colloid is key to the shift in absorption onset and ultimately the shift in *E*_g_. Said otherwise, the absorbance onset in the colloidal suspensions around 4 eV is independent of concentration or path length (Figure 2C) because, in the colloidal state, 1DLs are not interacting electronically, only physically, as shown by the liquid crystal (LC) behavior (see Figure 4 below). It is only upon drying that the 1D to 2D transition takes place, which results in the changes observed in Figure 2A.

At the onset of absorbance at ≈4.0 eV (≈312.5 nm), the *A* of the colloids, when normalized by *b*, exhibits linear Beer–Lambert behavior (Figure 2D). According to the Beer–Lambert law, an average mass absorption coefficient, ε̅_m_, can be extracted, assuming
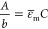
If this relationship is valid, then the slope of an *A*/*b* vs *C* plot (Figure 2D) is a measure of ε̅_m_. A linear fit of the data points in Figure 2D yields an ε̅_m_, at 312.5 nm, of 2.0 ± 0.4 Lg^–1^ cm^–1^. Knowing this value, the concentration of a given 1DL colloid can be determined from a simple absorbance measurement. We note that both Sasaki et al.^34^ and Serpone et al.^73^ showed Beer–Lambert behavior for aqueous LT suspensions and anatase nanoparticles, respectively. In short, 1DLs in colloidal form behave no different optically than any other colloidal SNs or light-absorbing compounds in the literature.

**Figure 3:**
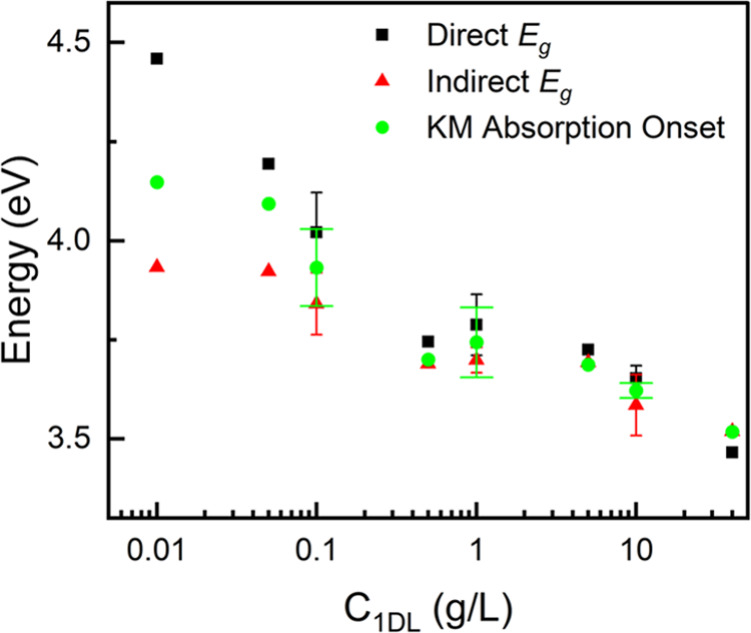
Energetic onsets or *E*_g_ values of 1DL filtered films as a function of concentration. Direct (black squares) and indirect (red triangles) *E*_g_ values, using the Tauc method, and KM treatment absorption onsets (green circles) calculated using the method established by Makuła et al.^68^ using adjusted baselines when necessary. Error bars shown are of standard deviation between 3 discrete 1DL batches. Raw solid-state UV–vis data on filtered films are available in Figures S4–S6, with KM and Tauc plots shown in Figures 2A,B and S4–S6.

Most papers in the 2D LT literature treat such materials as indirect semiconductors, with an *E*_g_ of ≈3.8 eV.^35,39,42^ However, more recent DFT work, and our results below, predict them to be direct semiconductors,^43−45^ hence our decision to present them as such in Figure 2B. However, to compare our results with others,’ as in Figure 3, we present the *E*_g_ values from treating the 1DLs as either direct (black squares in Figure 3) or indirect SNs (red triangles in Figure 3). A shift in the KM absorption onset (green circles in Figure 3) is also observed. The main takeaway from these results is that *E*_g_ increases substantially if the system is assumed to be direct. The goal of this work is not to sort this problem out but rather to bring it to the fore. Based on our and others’ DFT calculations, 1DLs will henceforth be treated as being direct SNs.

Since the film fabrication method used in this study is simply vacuum filtering, in general, the more concentrated the colloid, the thicker the film—which is obvious when looking at the films themselves (Figure S9)—and quantifiable in thickness measurements (Figure S10). According to some of the existing literature on the subject, the impact of TiO_2_ film thickness on *E*_g_ is minuscule, with variations no greater than 0.1 eV,^74,75^ especially for amorphous films.^76^ When comparing the KM absorption onsets as a function of film thickness (Figure S11), it is apparent that thicker films exhibit higher wavelengths of absorption onset (i.e., lower *E*_g_) than thinner ones. However, the fact that this trend is nonlinear is important since it indicates that film thickness is not the main variable impacting the optical properties of the 1DL films. There are studies that observe a more drastic shift in *E*_g_ (≈0.3 eV) with films of thicknesses on the order of 10s of nanometers^77^ and micrometers^78^—more akin to the thicknesses of the films in this study. However, these studies concluded that thicker films have wider *E*_g_ values, contrary to what is observed here.

### Liquid Crystal (LC) Phase

**Figure 4:**
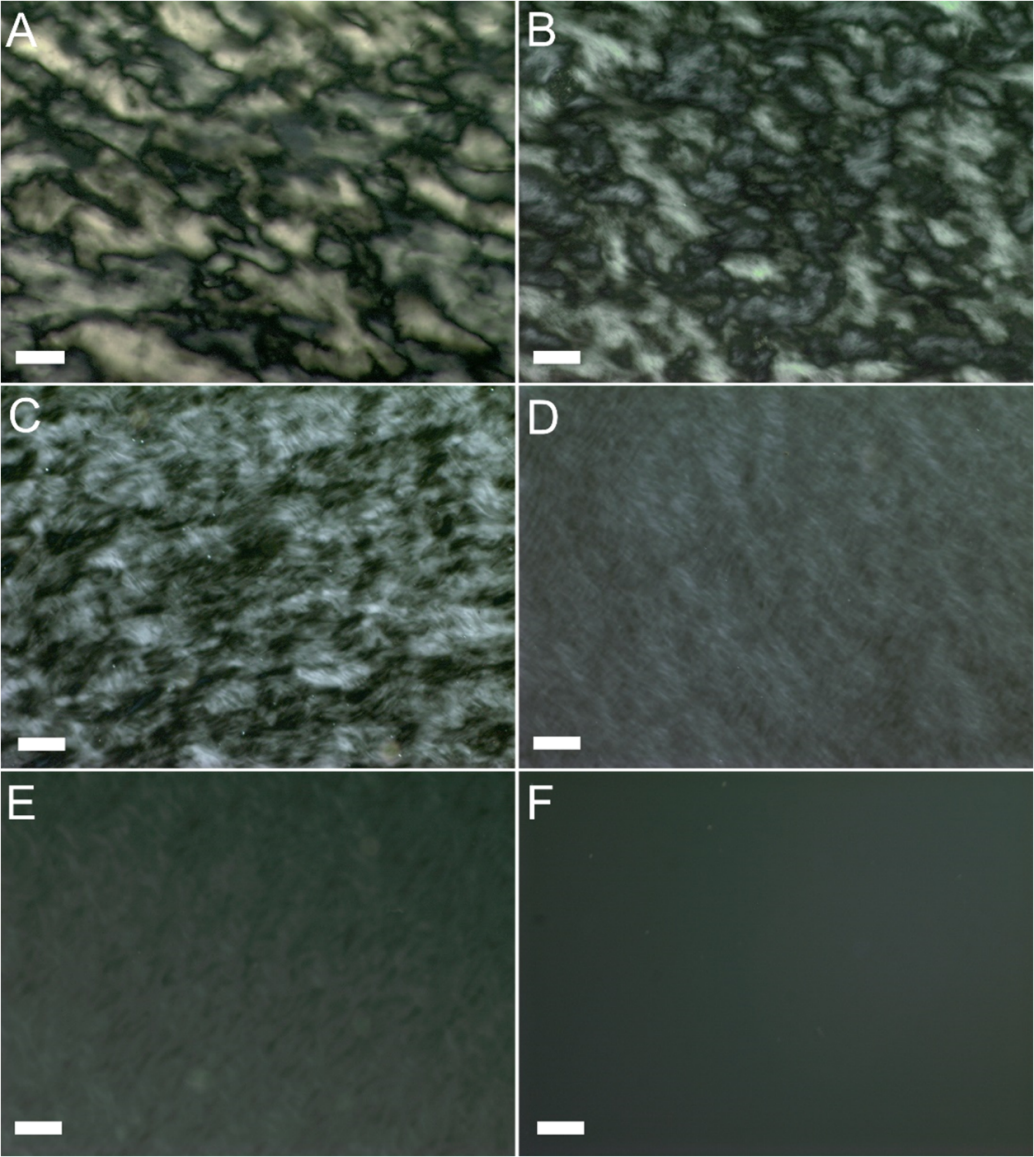
PLM micrographs of 1DL aqueous suspensions of various concentrations. (A) 40 g/L, (B) 20 g/L, (C) 10 g/L, (D) 4 g/L, (E) 3.5 g/L, and (F) 1 g/L. Images taken at 10× magnification; scale bars represent 100 μm. 1DL suspensions drop-cast on a glass substrate and imaged immediately prior to drying.

Based on the fundamental principles of collision theory, upon increasing colloid concentration, 1DLs are more likely to interact with each other and may do so in energetically favorable ways. To prove this important point, a series of colloidal suspensions were prepared at concentrations from 40 to 1 g/L. These were then imaged using a polarized light microscope (PLM). Figure 4 shows representative textures of the suspension. Schlieren texture can be seen in samples with concentrations from 40 to 10 g/L (Figure 4A–C), indicating that a nematic LC phase is formed in these suspensions. Weak birefringence can be seen for samples with concentrations between 10 and 3.5 g/L (Figure 4D,E). The birefringence disappears at a concentration <1 g/L (Figure 4F).

Materials that are anisotropic in shape are known to show lyotropic, or concentration-dependent, LC behavior at certain concentrations.^79,80^ For example, 1D materials, such as ZnO nanowires, Au and CdSe nanorod arrays, and carbon nanotubes, are inherently anisotropic due to their aspect ratios and readily form nematic and smectic LC phases with orientational order in various solvents.^81−86^ Due to the polydispersity of 1DLs, primarily with regard to their length, the critical LC concentration will probably vary based on sample and processing conditions. As a result, in general, we expect the critical concentration of the nematic to isotropic phases to be somewhere between 1 and 10 g/L.

### Structural Order Change

Given the evidence of a lyotropic LC phase, it is reasonable to conclude that at high concentrations, the 1DLs are sufficiently close enough to interact with each other physically. Furthermore, since the optical activity seemingly shifts from a more quantumly confined 1D material to 2D, it is reasonable to suspect these interactions may even result in Ti–O–Ti bonds along the *c*-direction upon drying. To assess the validity of this claim, Raman spectroscopy and powder X-ray diffraction (XRD) were employed to understand if there are structural changes. A transmission electron microscope (TEM) was also used to image the materials, but little useful information was obtained (see below).

**Figure 5:**
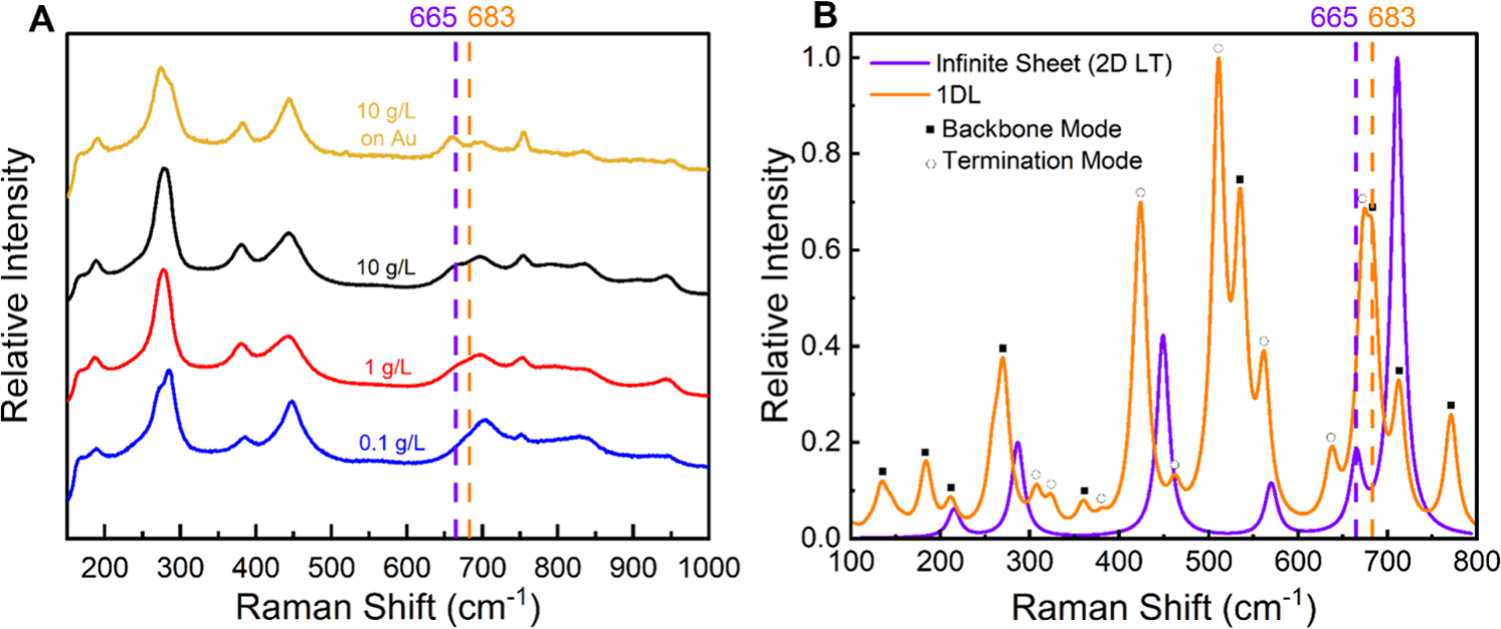
Raman spectra for 1D and 2D LTs. (A) Measured Raman spectra of films formed by vacuum filtering of noted concentrations of 1DL colloidal suspensions. Top spectrum (gold) shows a 10 g/L (black) sample drop-cast on a gold-coated Si wafer, resulting in SERS that exaggerates certain modes. (B) Calculated Raman spectra for 1DL and 2D LTs. Backbone (filled square) and termination (open circle) modes indicated over the 1DL spectrum. Spectra and labels are color-coded.

Colloids of various concentrations were filtered, and the Raman spectra of the resulting films were acquired (Figure 5A). While the Raman spectrum of each sample showcases bands similar to 2D LTs,^59^ the band around ≈665 cm^–1^ (purple dashed line in Figure 5A) is of particular interest. According to Hu et al.,^87^ this band originates from the same A_g_ mode as the ≈700 cm^–1^ band (orange dashed line in Figure 5A) and is associated with the bending of TiO_6_ octahedral layers, suggesting that its presence depends on the planar or 2D nature of the LT sheets. Interestingly, when the colloidal concentration is well below the LC range (0.1 g/L), the band at ≈665 cm^–1^ disappears or, at least, becomes much less prominent, and a slight broadening is observed at the 700 cm^–1^ band. The emergence of the ≈665 cm^–1^ band at concentrations around/above the LC range (10 g/L) was amplified by a surface-enhanced Raman scattering (SERS) effect.^88^ This was for a sample prepared by drop-casting the 10 g/L colloid onto a Si wafer with a 20 nm thick gold coating deposited by an electron beam (top spectrum in Figure 5A).

To better understand the structural origin of the Raman band’s disappearance around 665 cm^–1^, DFT calculations were carried out to simulate the phonon vibrations and Raman spectra of the base unit structure of 1DL (Figure 1) as well as an infinite 2D sheet of LT (Figure S12). The vibrational modes of individual Raman peaks are summarized in Table S1. Figure 5B presents the complete simulated Raman spectra, with the 1DL peaks categorized into two groups: those arising from the backbone Ti–O bonds (solid squares) and those from edge termination groups (open circles), i.e., –H, –OH, and H_2_O. The simulated Raman spectrum of the 2D LT is also in good agreement with the values reported in the literature.^89^ More interestingly, we note that the 665 cm^–1^ peak of 2D LT, which corresponds to an in-plane stretching of the Ti–O bonds along the *a*-axis, shifts to 682 cm^–1^ for the 1DL (see the dashed orange vertical line in Figure 5B), which is in good agreement with experimental observation in the disappearance of the former and the broadening of the latter observed when at low concentrations (Figure 5A). The importance of this observation cannot be overstated, as this is strong evidence that the 1DLs can bond themselves into 2D sheets observed in previous reports,^46,47,56,90^ when their concentration is high enough.

We note the presence of several simulated peaks in Figure 5B that are not in the experimental spectra in Figure 5A, in particular between 500 and 650 cm^–1^. Some of the peaks are attributed to the vibration modes associated with the termination groups at the edges of the 1DLs (Table S1). The 1DL (Figure 1) structural input is simply the 2D structure that is cut to 2 TiO_6_ octahedra in width, creating new “edges” with water molecules and their dissociated –H and –OH as terminations. In reality, these vibration modes could be largely broadened and/or shifted as the actual configurations of edge terminations are not as perfect and ordered as assumed by the DFT calculations. Moreover, these edges are not in the growth or *a*-direction but in the orthogonal *c*-direction. As mentioned throughout our previous work, we hypothesize that TMA^+^ is a templating agent attracted to the negative charge on the “new edges” which would, in practice, create electrostatic interactions with TMA^+^ that would dramatically reduce the bond polarizability of the associated Ti–O stretch. In summary of this section, the “edges” generated by “cutting” the 2D LT to create the 1D structure produce additional vibrational modes in the DFT model because they are not capped with TMA^+^, and so the edge Ti–O stretch may be misrepresented compared to experimental. The backbone modes (Table S1) that are absent in the experimental data could be the result of the assumption that Raman bands are nonresonant, a theoretical limitation of DFT, or due to the calculations being done with both systems being in the vacuum state.

An additional observation is that the mode at ≈190 cm^–1^ seems unique to the 1D calculated structure, the experimental results, and to the LT literature.^59^ Importantly, this mode is present in all experimental Raman spectra in this work, regardless of the concentration, and in our previous work.^47^ This mode corresponds to complex, in-plane vibrations of the Ti–O backbone, as described in Table S1. In contrast, the 190 cm^–1^ mode is absent in the 2D LT calculations, and another low-intensity mode at ≈215 cm^–1^ appears, which corresponds to the in-plane stretching of the Ti–O backbone along the *a*-direction.

To better understand the structures at the atomic level, TEM micrographs of each concentration were obtained and are shown in Figure S13. Unfortunately, they were inconclusive as to the nature of what was being imaged due to the thicknesses of the flakes observed. In most cases, 1DLs need to be diluted heavily (≪10^–4^ g/L) to produce samples thin enough for TEM analysis, far lower than the concentrations used in this study.^46^ It is important at this juncture to note that the SAED of most of the TEM micrographs indicated that they were TEM amorphous (Figure S13). The simplest explanation here is that the flakes are composed of tangles of 1DLs and assemblies of the same, perhaps from the dropcasted evaporation compared to vacuum assisted filtration, consistent with more detailed work on the matter.^56^

**Figure 6:**
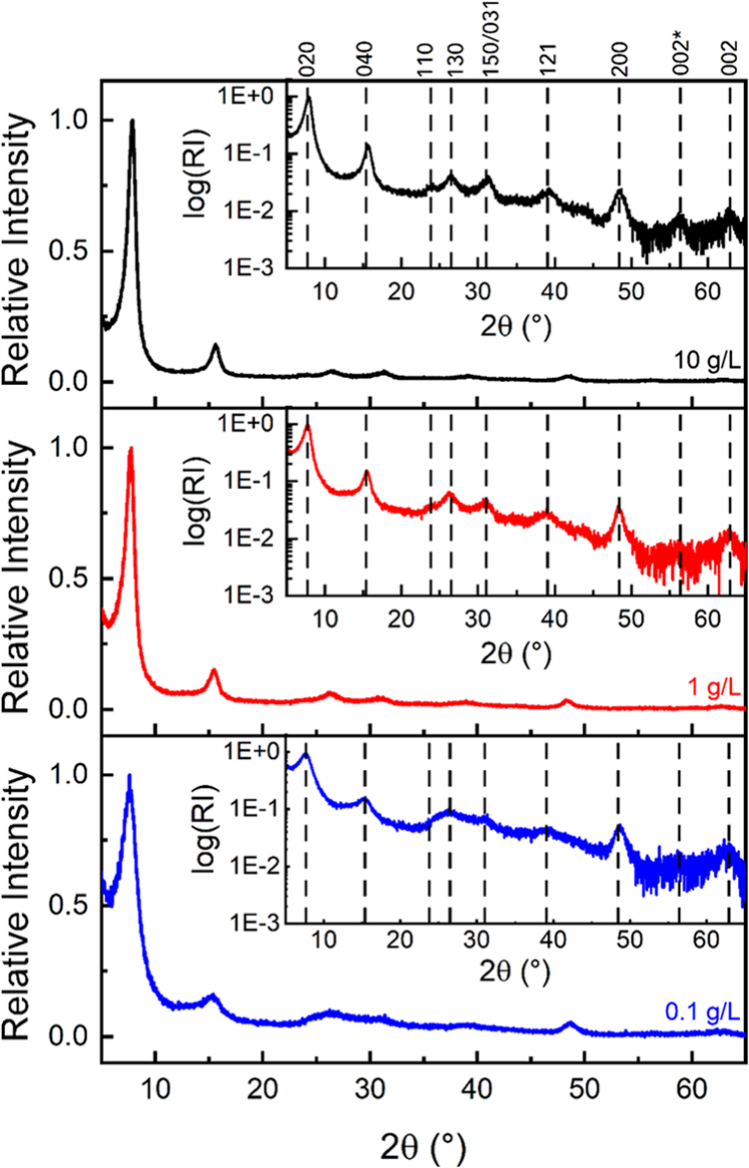
Effects of colloidal suspension concentrations on XRD characteristics of finely powdered filtered films. XRD patterns of finely crushed films with a prefilter concentration of (top) 10 g/L, (middle) 1 g/L, and (bottom) 0.1 g/L. Insets show same results on a semi-log plot and include the indices of the peaks (shown on top of the top panel) as to our latest understanding.^53^

To further probe the structures of our filtered films, we obtained their XRD patterns at the three different concentrations indicated in Figure 6. Table 1 lists the XRD peak characteristics and Scherrer equation^91^ results for the 020 (2θ ≈ 7.5°) and 002 peaks (2θ ≈ 63°). The 002 peaks are useful in determining the crystallite size in the *c-*direction, or filament width. The corresponding values of the 200 peaks (2θ ≈ 48°) are given in Table S2. The results for the 020 peaks are plotted in Figure S14. To enhance the signal-to-noise ratio for the 002 peaks, slow scan rate XRD patterns were obtained in the 60–65° 2θ range (Figure S15).unambiguous evidence for widths along c.

**Table 1:** XRD Peak Characteristics and Scherrer Equation Results Based on 020 and 002 Peaks Calculated from Data Shown in Figures 6 and S15, Respectively

	020 peak or *b*-direction				002 peak or *c*-direction			
[1DL] (g/L)	2θ (deg)	*d* (Å)	fwhm (deg)	crystallite size (nm)	2θ (deg)	d (Å)	fwhm (deg)	crystallite size (nm)
10	7.88	11.2	0.015	9.2	62.48	1.5	1.52	6.1
1	7.76	11.4	0.016	8.7	62.68	1.5	1.45	6.4
0.1	7.62	11.6	0.024	5.9	62.66	1.5	1.79	5.2

From these results, we conclude that changing the 1DL concentrations over 2 orders of magnitude does not greatly directly impact order. For example, in the case of *b*-direction, the crystallite size along the stacking direction increases from ≈6 nm at 0.1 g/L to ≈9 nm at 10 g/L. At ≈9 nm, the crystallite size along the *b*-direction of the 10 g/L sample is consistent with our previously published work.^56^ In the 002 and 200 cases, the variations, if any, are even smaller. The changes in *d*-spacings, on the other hand, were not a function of colloid concentrations in the 002 and 200 cases. The changes in the 020 case are modest but appear to scale with the logarithm of the concentrations (Figure S14). The simplest explanation is that upon dilution, the TMA^+^ or the 1DLs themselves are better hydrated and remain that way through drying.

The domain sizes along 002 are consistent with those of 1DL films measured in a recently published TEM study.^57^ It must be mentioned that even at a width of 5.2 nm, the 1DLs are not close to the accepted range of exciton radii for anatase TiO_2_,^73^ and, to the authors’ knowledge, there is no work on excitonic radii for LTs. There is, however, theoretical work detailing that exciton radii are directional in anatase, and that may be the case for LTs as well.^92^ This investigation will be the subject of future work.

In the 2θ ≈ 56° region of the XRD patterns, a new peak appears that is present only in the most concentrated samples (Figure 6). This peak is denoted 002* and is hypothesized to be due to the formation of a new Ti–O–Ti system, where the center-to-center distance of the Ti atoms is ≈3.2 Å. Since this peak occurs only in the samples with concentrations above the LC range, it is likely indicative of interfilament bonding, a claim that is further supported by the Raman data (Figure 4). However, it cannot be discounted that this peak could also correspond to (1 10 1), as illustrated in the published DFT-calculated XRD pattern of 1DLs.^57^

### Calculated Electronic Band Structures

**Figure 7:**
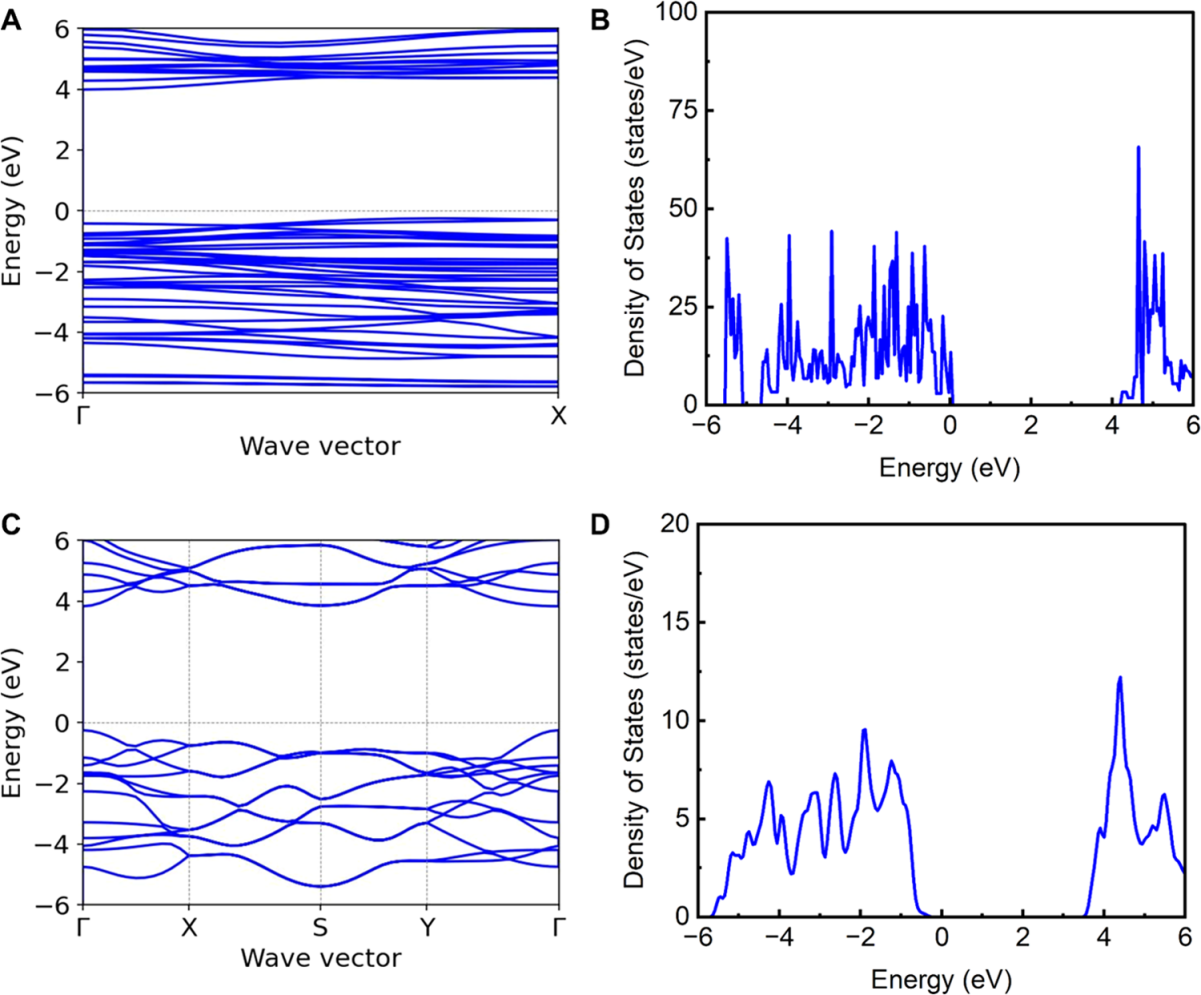
DFT-calculated electronic properties of 1DL and 2D LT. (A) Electronic band structure and (B) DOS of 1DL, shown in Figure 1. (C) Electronic band structure and (D) DOS of 2D LT (Figure S12). Both (A) and (C) were calculated as single units in vacuum.

To shed further light on the effects of the 2D-to-1D dimensionality transition on increasing *E*_g_, DFT calculations were performed to predict the electronic band structures of the unit structure of 1DL (Figure 1) and 2D LT (Figure S12). Hybrid HSE06 functionals were employed to describe the exchange–correlation behavior of electrons for seeking a higher prediction accuracy. The calculated electronic band structure and density of states (DOS) are shown in Figure 7 for the 1D and 2D structures, respectively. 2D structures exhibit a direct *E*_g_ (Figure 7C), which is consistent with literature DFT results on 2D LTs.^43−45^ The 1D structures, on the other hand, exhibit an indirect band gap (Figure 7A). *E*_g_ of the 1DL was predicted to be 4.24 eV (Figure 7B), higher than that of the 4.08 eV *E*_g_ for 2D LTs (Figure 7D). This finding qualitatively supports the argument that quantum confinement could be a major driver for the experimentally observed *E*_g_ in the colloid samples with different concentrations. However, we also acknowledge that there is a large discrepancy between the theoretically predicted *E*_g_ shift (0.16 eV) and the ∼1 eV experimentally observed (Figure 3). As discussed above, the actual 1DL atomic structure is most probably much more complex than the simplified model used for DFT calculations here, where the contributions from TMA^+^ and *b*-direction stacking are not considered, which may explain the discrepancy. Due to the implications of this behavior, it will be subject to further experimentation and calculations in the near future.

## Conclusions

We show that we can vary the *E*_g_ of filtered films from ≈3.5 to ≈4.5 eV by diluting the concentration of the aqueous 1DL colloid from 40 to 0.01 g/L, which is then filtered to produce the films. This wide range in *E*_g_ reinforces the conclusion that quantum confinement to 1D is important in 1DLs. The most important takeaway from this work is that the 1DLs, no matter the concentration, do not interact with each other electronically while in the colloidal state. However, once dried, the assembly is forced based on the colloid concentration , and the resulting structure thereby governs the electronic properties of the filtered films. The fact that the filtered films maintain a “history” of the collidal suspension they were made from is an imporant result since it allows for a simple method to control *E_g_.* This simple control of dimensionality, and ultimately *E*_g_, is just another demonstration of the exceptional properties of 1DLs.

## Methods

### 1DL Colloid Fabrication

10 g of titanium diboride (as-received 99.9%, −325 mesh, Thermo Fisher Scientific Inc., Waltham, MA) was added to 87.5 g of tetramethylammonium hydroxide (as-received 25% w/w aqueous 99.9999% pure, Alfa Aesar, Ward Hill, MA) in a 250 mL high density polyethelyne, HDPE, bottle vented with a single 23-gauge needle. The bottle was heated and shaken in an incubator (Labnet International Shaking Incubator, NJ) at 200 rpm and 80 °C for 4 days. The resulting sediment was combined with ethanol (as-received, 200 Proof, Decon Laboratories Inc., King of Prussia, PA), vortex shaken, and centrifuged at 3500 rpm for 2 min. The clear supernatant was discarded after each wash. This was repeated until a pH ∼ 7 was achieved (usually 3 times), measured using pH strips. Ultrapure water (<18.2 mΩ/cm) was added to the ethanol-washed product, and the material was suspended by vortex mixing. The mixture was centrifuged at 5000 rpm for 1 h, resulting in a highly stable 1DL pure colloidal suspension as any unreacted powders settled to the bottom.

After processing the concentrations of the colloidal 1DL suspensoins were in the range of ≈40 g/L. This concentration was confirmed by vacuum filtering 2 mL of colloidal 1DL through a 25 μm thick microporous monolayer polypropylene membrane (Celgard 3501, Celgard, NC) over a fritted glass filter apparatus. Once filtered, the solid was dried in an oven, under vacuum, at 80 °C, and the weight of the residue was measured.

### Colloidal State Characterization

The light absorption behavior of the 1DL colloids, shown in Figure 2, were recorded using a Cary 60 UV–vis spectrophotometer (Agilent Technologies, CA) with a scan rate of 300 nm/min. The scans were taken using quartz cuvettes with the noted path lengths (1 cm and 1 mm).

The PLM images shown in Figure 4 were taken with a BX51 optical microscope, OM (Olympus, Tokyo, JP). The orientation of the polarizer was normal to the analyzer for all images. Images were acquired with a Diagnostic Instruments SPOT Insight model 3.2.0 camera using SPOT advanced image acquisition software. For sample preparation, 20 μL of 1DL colloid was cast onto glass slides pre-cleaned with isopropanol. Thickness control was achieved using a poly(ethylene) spacer with a thickness of ≈34 μm. After depositing the droplet of 1DL colloid, a coverslip was gently placed onto the droplet. The samples were stored in the dark at ambient conditions to minimize water evaporation.

### Film Formation (for Solid-State Characterization)

Films were formed by adding 1DL colloid of various concentrations and vacuum filtered over a fritted glass filter apparatus to dry (with Celgard 3501). The films were then allowed to dry fully in a fume hood for at least overnight before they were characterized. For all-solid-state UV–vis and Raman measurements, the films were used as-filtered. For powder XRD measurements, the films were finely crushed using an agate mortar and pestle. For TEM and SERS, the colloids were directly drop-cast on a graphene-reinforced lacey carbon grid and gold-coated Si wafer, respectively.

### Solid-State Characterization

The band gap measurements shown in Figure 2, both direct and indirect, were generated by acquiring solid-state UV/vis profiles for each sample and evaluating the resulting data by the KM and Tauc methods. The evaluated samples were prepared as thin films by filtering a variable volume (depending on the concentration) of the colloid onto Celgard such that ∼100 mg of 1DL was present in each case, regardless of the original concentration. Measurements were acquired using a dual beam UV/vis spectrophotometer (UV-3600i Plus Spectrophotometer, Shimadzu, Kyoto, Japan) equipped with a 150 mm diameter integrating sphere attachment (ISR-1503, Shimadzu, Kyoto, Japan). The films were kept on Celgard when placed on the BaSO_4_-coated sample holder, which was loaded into the integration sphere. A blank piece of Celgard was also placed on the sample holder for the reference beam. Prior to each test, a profile was acquired for a blank piece of Celgard in both the sample and reference beam paths to ensure that a proper baseline was established. Each thin film sample was scanned across the wavelength range of 200–800 nm, and the % reflectance was recorded. The resulting raw data was processed in OriginPro.

To acquire the Raman spectra of the samples shown in Figure 5A, a small section of the film was loaded onto a glass microscope slide. An RM-2000 Vis Raman spectrometer (Renishaw, Wotton-under-Edge, England, U.K.) was used to probe the observed Raman shifts in each sample using a 633 nm beam source. The samples were exposed to 100% laser power from wavenumbers of 150–1000 cm^–1^. Due to an incited phase change as the result of previous work,^46,47^ the spectra were acquired at 25% laser power before, and after, the 100% scan to confirm no phase change occurred. This was indeed the case.

Powder XRD patterns were acquired with a benchtop XRD instrument (MiniFlex 600, Rigaku, Tokyo, Japan) equipped with a Cu–K_α_ radiation source. Patterns shown in Figure 6 were scanned from 3 to 65° 2θ with step increments of 0.02 s^–1^ and a 1 s hold time. To better delineate the 002 peak, slow scan patterns, shown in Figure S15 were scanned from 60 to 65° 2θ, with step increments of 0.02 s^–1^ and a 4 s hold time.

Film thickness measurements were taken using a Keyence VK-X3000 Series 3D surface profiler (Figure S10).

TEM images shown in Figure S13 were obtained by using a 2100F field emission TEM (JEOL, Ltd., Tokyo, JP).

### DFT Calculations

The DFT calculations were performed using the Vienna Ab initio Simulation Package (VASP).^93−97^ The projector augmented wave method (PAW)^97,98^ was employed with an energy cutoff of 600 eV for the plan-wave basis set. The VASPKIT package was used to assist with pre- and postprocessing of the calculations.^99^ The exchange–correlation interactions of electrons were described using the generalized gradient approximation (GGA) functional developed by Perdew–Burke–Ernzerhof (PBE)^100^ for the calculations of structure relaxation and phonon spectra, while the hybrid HSE06 functionals^101,102^ were employed for the electronic band structure calculations. The mixing fraction of the Hartree–Fock exchange in the hybrid functionals was calibrated to correctly reproduce the experimental band gap values of bulk anatase. The energy convergence criterion of the electronic self-consistency was set as 10^–7^ eV for the relaxation and electronic band structure calculations, while the ionic relaxation was set to stop when the Hellmann–Feynman force on each atom is smaller than 0.001 eV/Å. The Brillouin-zone integration of the electronic band structures were carried out using the Gaussian-smearing method with a width of 0.1 eV. The density functional perturbation theory (DFPT) and finite-displacement methods were employed for the phonon calculations and analyzed using the Phonopy package.^103^ Based on the phonon calculation results, the Raman spectra were simulated using the Phonopy-Spectroscopy package.^104^

A slab model, with periodicity along the a- and c-directions, was used to construct the simulation supercell for 2D LT. A vacuum region of 12 Å was added along the b-direction (stacking direction) to minimize, interactions between the 2D sheet and its periodic images. To construct the atomic structure of the 1DL unit base, a 2D LT sheet was sliced to result in 1D an atomic chain with the periodicity along the a-direction (growth direction) and a width as two times the *c*-lattice parameter of 2D LT (Figure 1A,B). The Ti atoms at the edges of the 1D chain with dangling bonds were passivated with H_2_O molecules or their dissociated –H and –OH groups to preserve the 6-fold octahedral coordination. This results in an overall stoichiometry of (TiO_2_)_1/2_(H_2_O)_1/2_ for the 1D structure. The 1D chain was then embedded in a supercell with vacuum regions along the b and c directions for performing the DFT calculations.

## Supporting Information Available

Untreated UV–vis data for both solids and colloids, film images and thickness measurements, DFT-calculated Raman mode assignments and schematics, TEM analysis, and further XRD analysis (PDF)
