# Supplementary material for: Concentration-Dependent Control of the Band Gap Energy of a Low-Dimensional Lepidocrocite Titanate
Source: ACS Nano. 2025 Jan 16;19(4):4855–66. doi: 10.1021/acsnano.4c16410 (PMC11803914; doi:10.1021/acsnano.4c16410)
Supplement: Supplementary file 1 — nn4c16410_si_001.pdf [file nn4c16410_si_001.pdf]

Supplemental Information for:

Concentration-Dependent Control of the Band Gap Energy of a Low-Dimensional Lepidocrocite Titanate

Adam D. Walter‡, Gregory R. Schwenk‡, Yuanren Liu, David Bugallo Ferron, Jeffrey T. Wilk, Lucas M. Ferrer, Christopher Y. Li, Yong-Jie Hu, Michel W. Barsoum*.*

‡These authors contributed equally.

**Corresponding authors. Lead correspondence: barsoumw@drexel.edu*

Table of Contents

Figures

Figure S1. Schematic of 1DL colloid fabrication process. Details outlined in main paper. 2

Figure S2. Photograph of a 1 g/L 1DL colloidal suspension. Laser is used to demonstrate Tyndall scattering, or Tyndall effect, which is a characteristic of colloidal suspensions. 2

Figure S3. Polyhedral view of the 1DL structure. (A) a-c plane, (B) b-c plane, and (C) a-b plane with TMA^+ cations in the interplanar space. Protons are excluded for simplicity. Circle around TMA^+ cations indicates the hydration shell based on molecular dynamics simulations[1] 3

Figure S4. Optical properties of 1DL batch (replicate) 1. (Upper-Left) Raw reflectance, (Upper-Right) Kubelka-Munk (KM) treated, (Lower-Left) Direct Tauc treated, and (Lower-Right) Indirect Tauc treated data. KM and direct Tauc plots for 10, 1, 0.1, 0.01 g/L are shown in **Figure 2A** and **2B**, respectively 3

Figure S5. Optical properties of 1DL batch (replicate) 2. (Upper-Left) Raw reflectance, (Upper-Right) Kubelka-Munk (KM) treated, (Lower-Left) Direct Tauc treated, and (Lower-Right) Indirect Tauc treated data. 4

Figure S6. Optical properties of 1DL batch (replicate) 3. (Upper-Left) Raw reflectance, (Upper-Right) Kubelka-Munk (KM) treated, (Lower-Left) Direct Tauc treated, and (Lower-Right) Indirect Tauc treated data. 5

Figure S7. Raw absorbance spectra of 1DL colloidal suspensions, measured in transmission. Path length and concentrations are detailed as figure titles shown in top and legends, respectively. Legends and graphs are color coded. 6

Figure S8. Plot of α vs. energy over a wider range than those shown in Figure 2C. 6

Figure S9. Photograph of 1DL films prepared for solid-state UV-Vis measurement. Colloidal concentrations (left to right) 10 g/L, 1 g/L, and 0.1 g/L. 7

Figure S10. Thickness profiles of 1DL films utilized in this study. Films from Batch 2 (**Figure S5**). 7

Figure S11. KM absorption onset as a function of film thickness. Films from Batch 2 (**Figure S5**). 8

Figure S12. 2D LT structure used for all calculations. Structure file available upon request. 8

Figure S13. TEM micrographs and associated SAED patterns for various 1DL colloids drop cast onto TEM grids. (A) 10 g/L, (C) 1 g/L, (E) 0.1 g/L, and (G) 0.01 g/L colloids. Inset in (C) shows area of high order in the center of the zoomed-out image. SAED patterns of regions associated with, (B) 10 g/L, (D) 1 g/L, (F) 0.1 g/L, and (H) 0.01 g/L. 14

Figure S14. Analysis of 020 XRD peak shown in Figure 6. Semi-log plot of (left axis, black) crystalline sizes in *b*-direction using the Scherrer equation[2] and (right axis, red) *d*-spacing associated with (020) peaks. 15

Figure S15. Slow-scan XRD patterns of the high angle (2Θ = 60-65°) region of finely crushed 1DL filtered films. Data utilized for Scherrer equation analysis presented in **Table 1**. 16

Tables

Table S1. Vibration modes of theoretical Raman spectra of 1DL and 2D LT. 8

Table S2. XRD peak characteristics and Scherrer equation results. Based on 200 peak calculated from data in **Figure 6**. 15

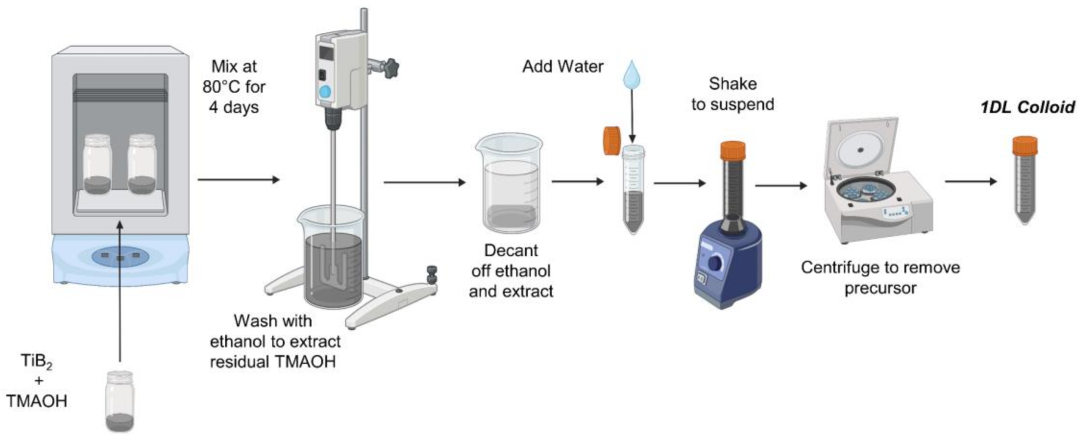

Figure S1. Schematic of 1DL colloid fabrication process. Details outlined in main paper.

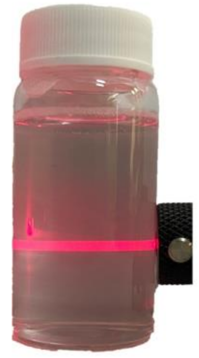

Figure S2. Photograph of a 1 g/L 1DL colloidal suspension. Laser is used to demonstrate Tyndall scattering, or Tyndall effect, which is a characteristic of colloidal suspensions.

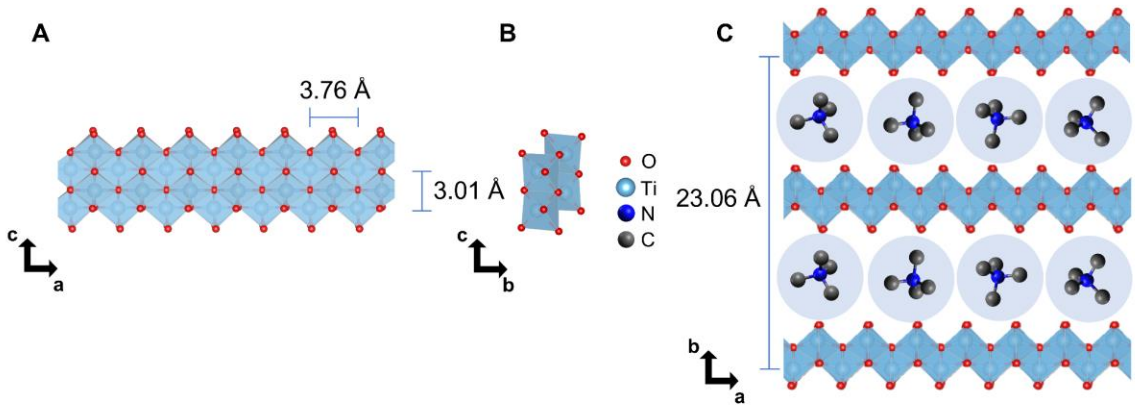

Figure S3. Polyhedral view of the 1DL structure. (A) a-c plane, (B) b-c plane, and (C) a-b plane with TMA^+ cations in the interplanar space. Protons are excluded for simplicity. Circle around TMA^+ cations indicates the hydration shell based on molecular dynamics simulations[1].

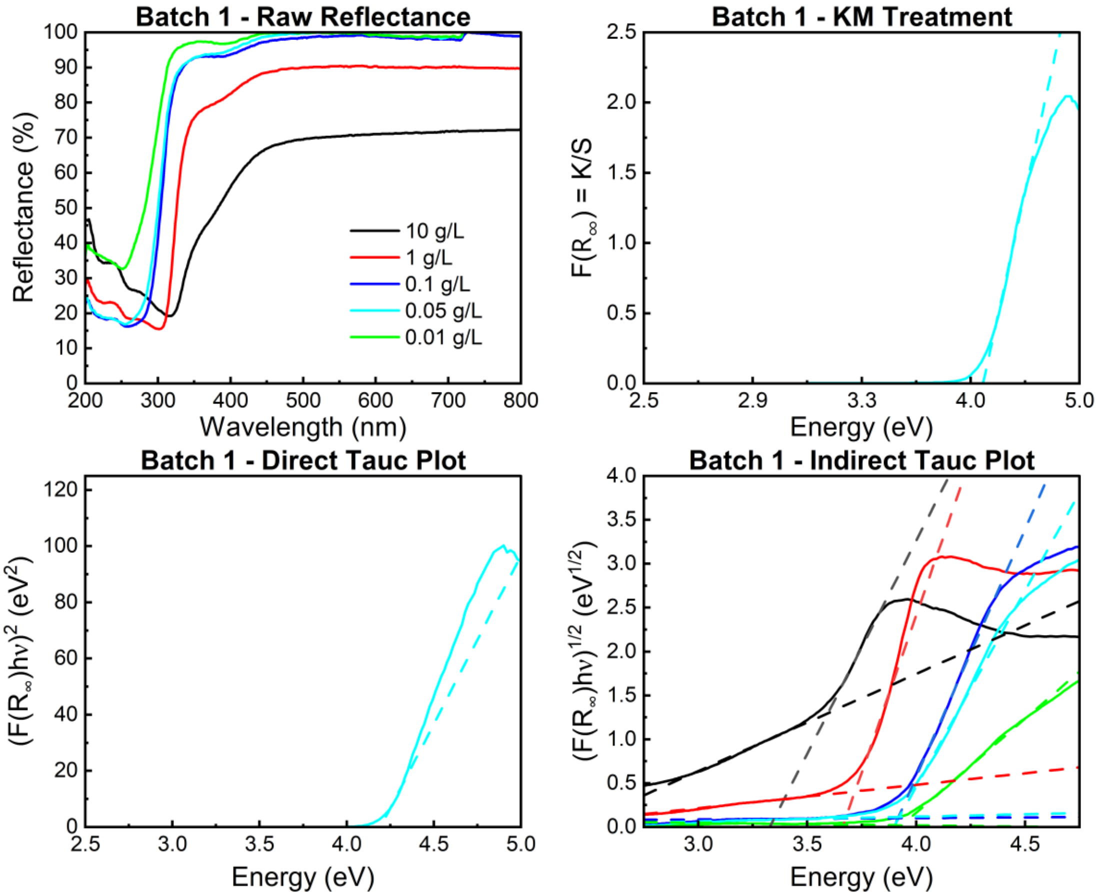

Figure S4. Optical properties of 1DL filtered films from batch (replicate) 1. (Upper-Left) Raw reflectance, (Upper-Right) Kubelka-Munk (KM) treated, (Lower-Left) Direct Tauc treated, and (Lower-Right) Indirect Tauc treated data. KM and direct Tauc plots for 10, 1, 0.1, 0.01 g/L are shown in **Figure 2A** and **2B**, respectively.

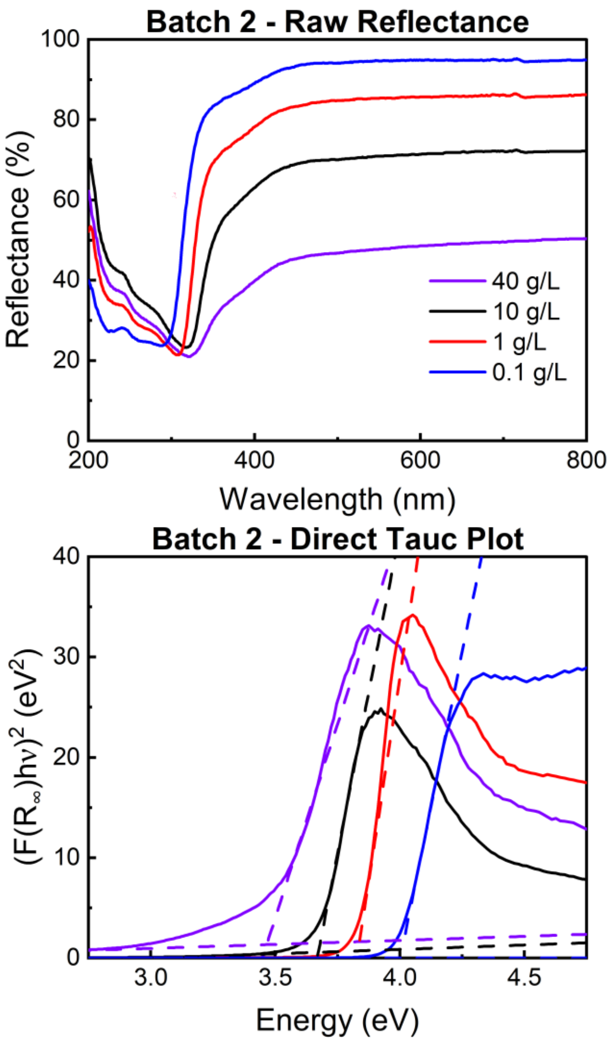

Figure S5. Optical properties of 1DL filtered films from batch (replicate) 2. (Upper-Left) Raw reflectance, (Upper-Right) Kubelka-Munk (KM) treated, (Lower-Left) Direct Tauc treated, and (Lower-Right) Indirect Tauc treated data.

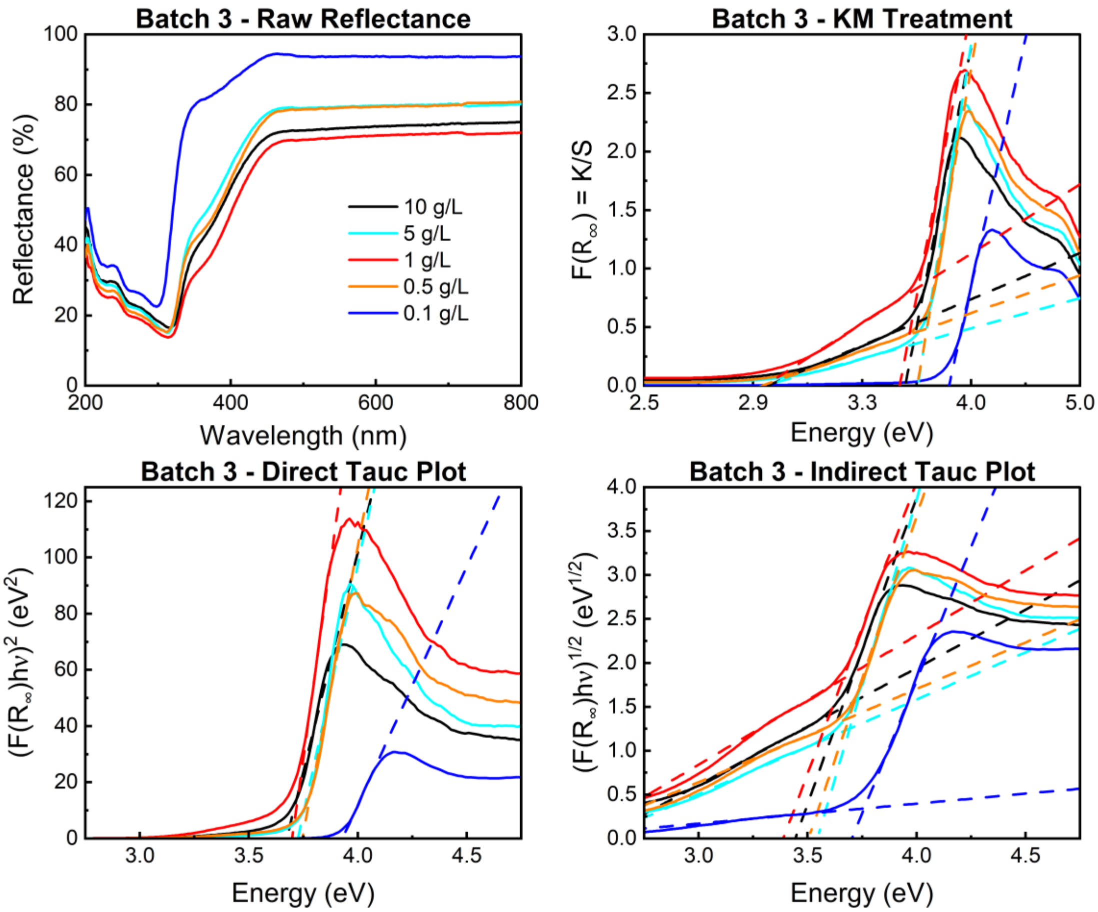

Figure S6. Optical properties of 1DL filtered films from batch (replicate) 3. (Upper-Left) Raw reflectance, (Upper-Right) Kubelka-Munk (KM) treated, (Lower-Left) Direct Tauc treated, and (Lower-Right) Indirect Tauc treated data.

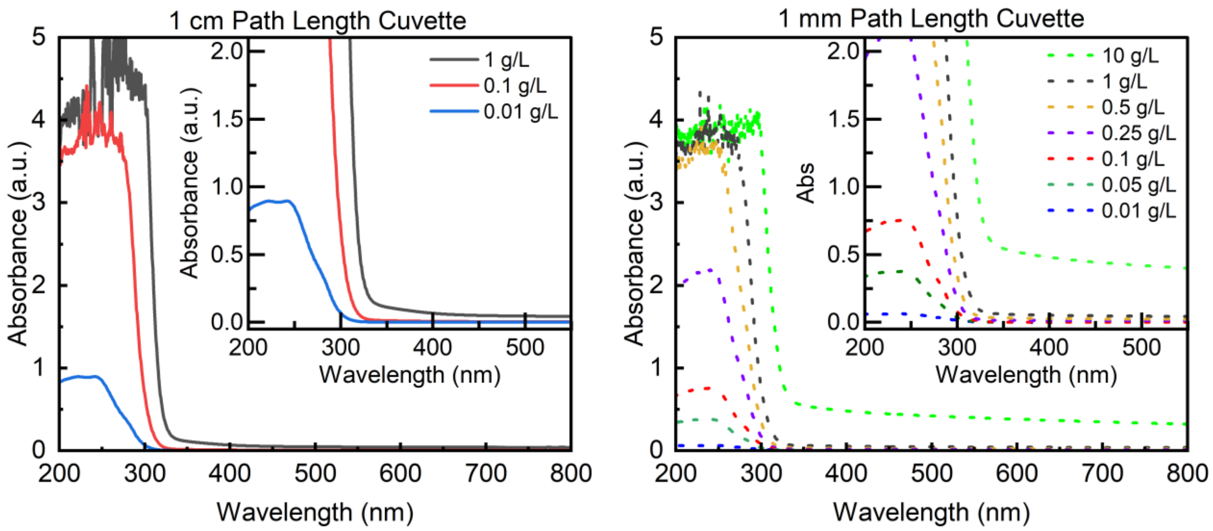

Figure S7. Raw absorbance spectra of 1DL colloidal suspensions, measured in transmission. Path length and concentrations are detailed as figure titles shown in top and legends, respectively. Legends and graphs are color coded.

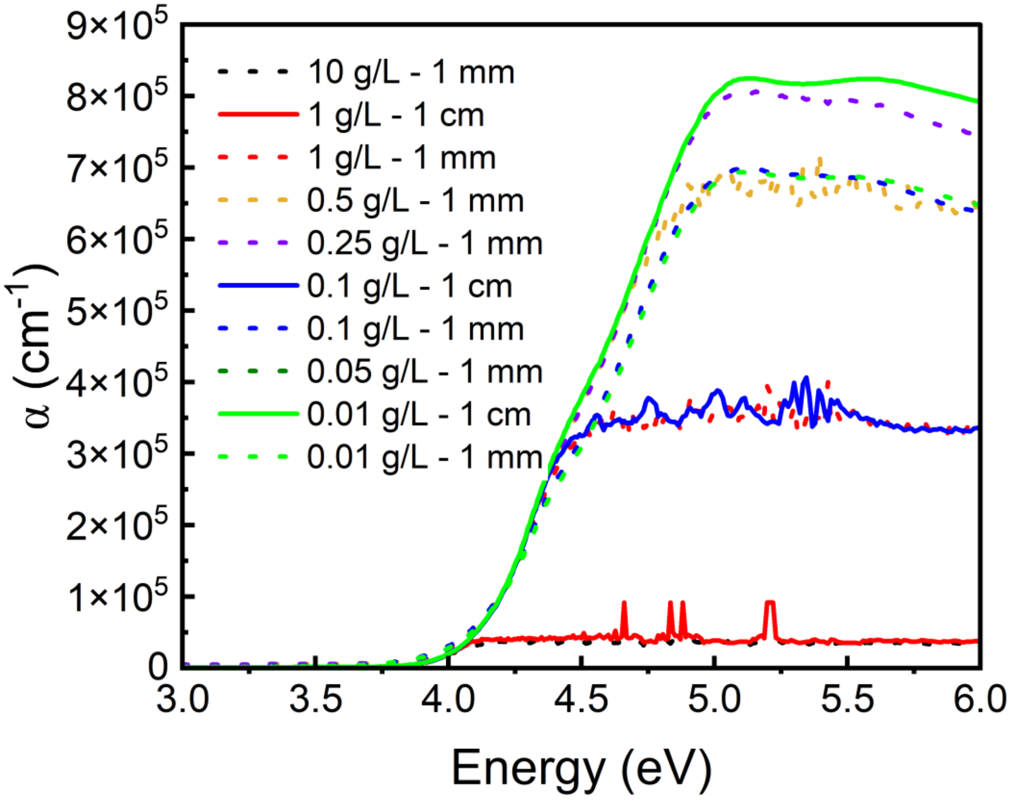

Figure S8. Plot of α vs. energy over a wider range than those shown in Figure 2C.

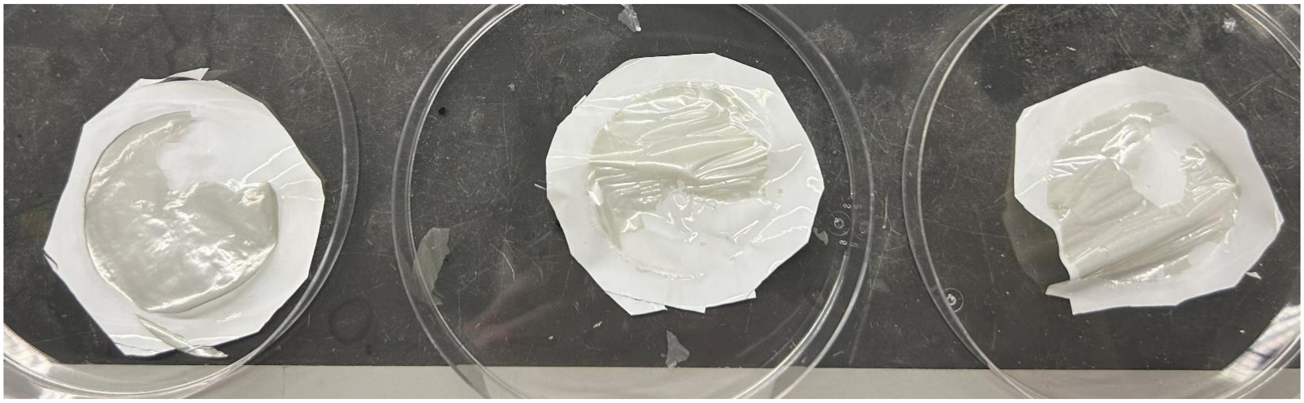

Figure S9. Photograph of 1DL filtered films prepared for solid-state UV-Vis measurement. Colloidal concentrations (left to right) 10 g/L, 1 g/L, and 0.1 g/L.

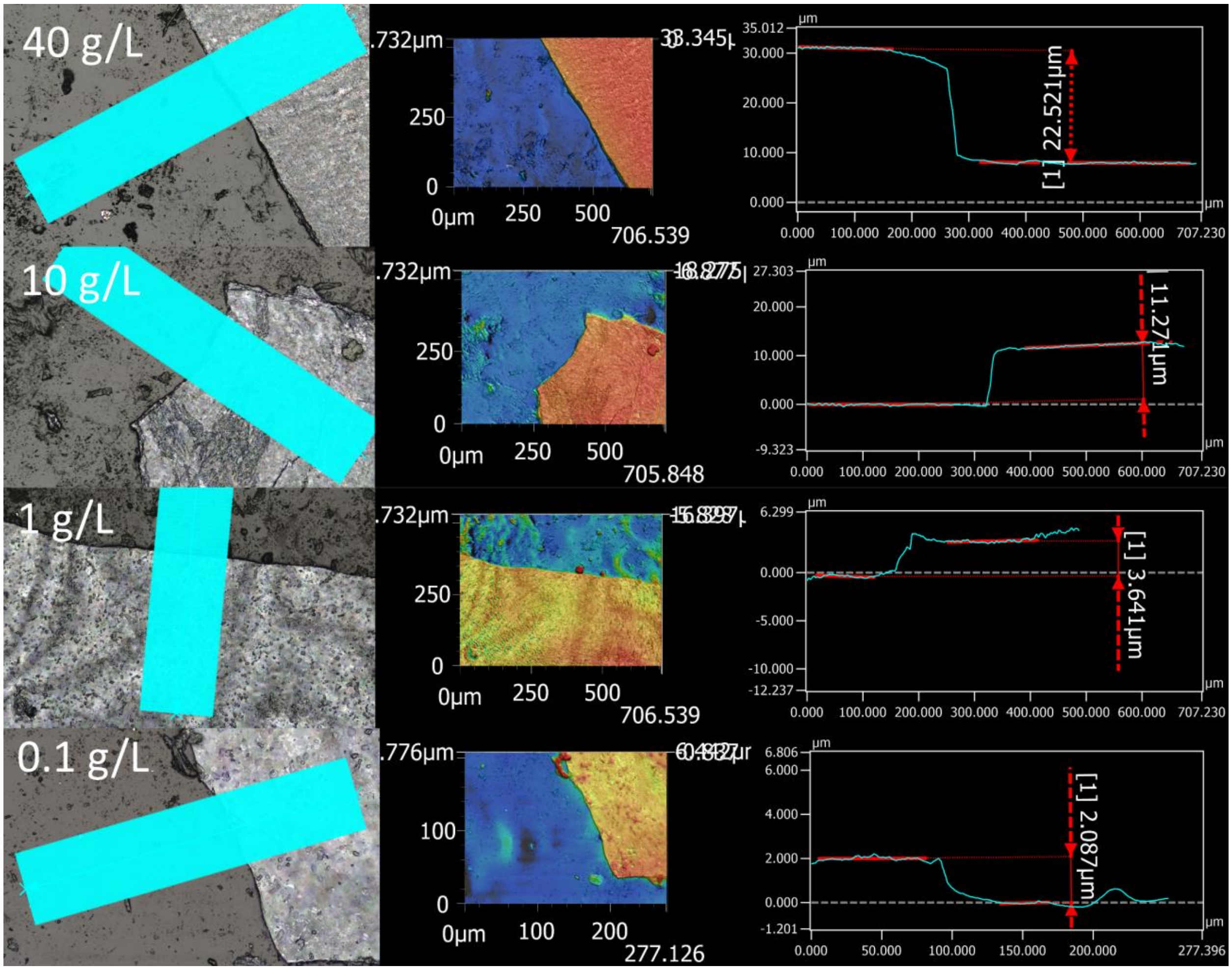

Figure S10. Thickness profiles of 1DL filtered films utilized in this study. Films from Batch 2 (**Figure S5**).

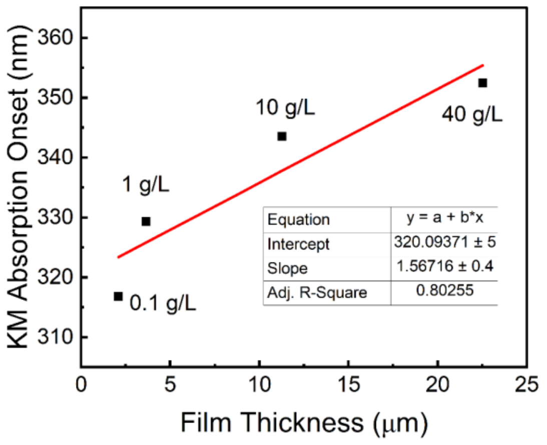

Figure S11. KM absorption onset as a function of film thickness. Films from Batch 2 (**Figure S5**).

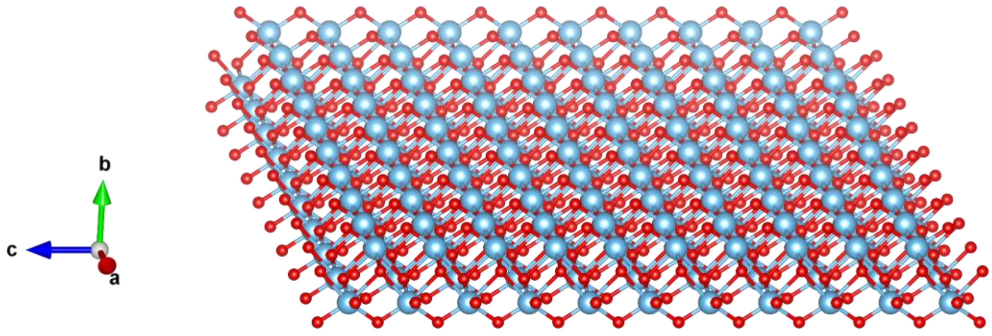

Figure S12. 2D LT structure used for all calculations. Structure file available upon request.

Table S1. Vibration modes of theoretical Raman spectra of 1DL and 2D LT.

Peak Position (cm^{-1})	Structure	Vibration origins	Vibration modes
771	1DL	Backbone (Ti-O)	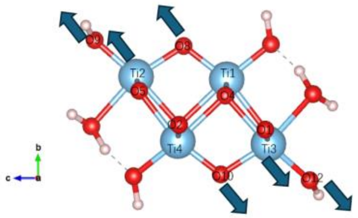

713	1DL	Backbone<br>(Ti-O)	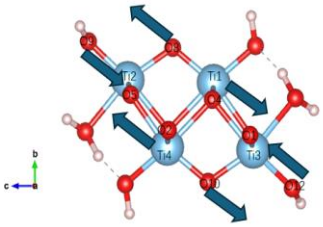
711	2D LT	Backbone<br>(Ti-O)	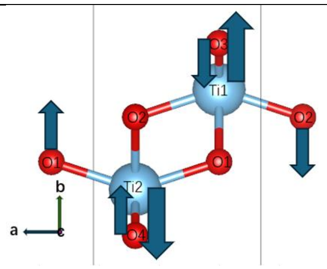
683	1DL	Backbone<br>(Ti-O)	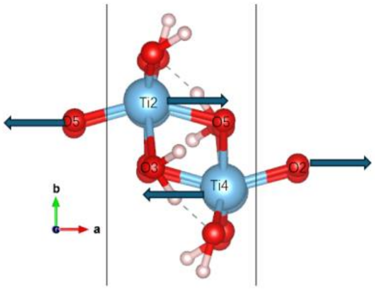
672	1DL	Termination<br>(-H, -OH, or H_2O)	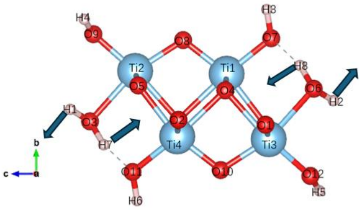 Minor vibrations also spread along a axis
665	2D LT	Backbone<br>(Ti-O)	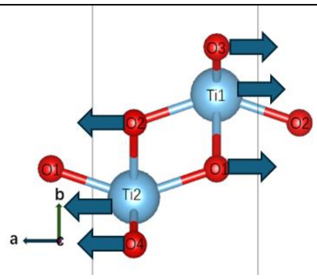

637	1DL	Termination<br><br>(-H, -OH, or H_2O)	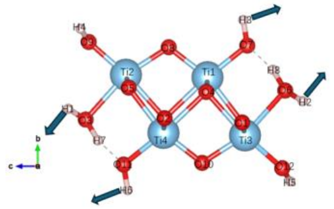<br>Minor vibrations also spread along a axis
570	2D LT	Backbone<br><br>(Ti-O)	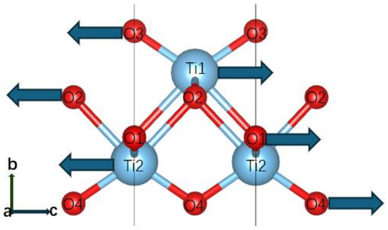
562	1DL	Termination<br><br>(-H, -OH, or H_2O)	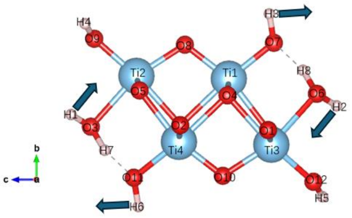
535	1DL	Backbone<br><br>(Ti-O)	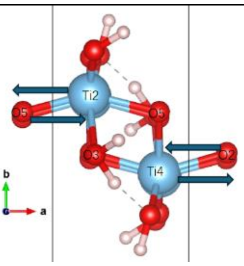
511	1DL	Termination<br><br>(-H, -OH, or H_2O)	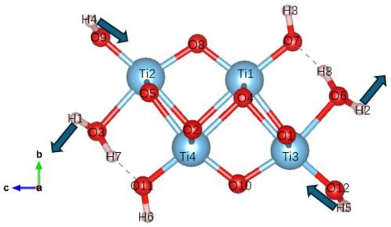<br>Minor vibrations also spread along a axis

462	1DL	Termination<br>(-H, -OH, or H_2O)	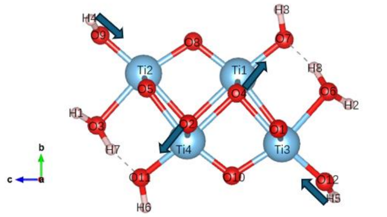<br>Minor vibrations also spread along a axis
450	2D LT	Backbone<br>(Ti-O)	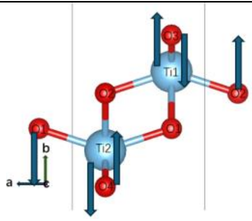
424	1DL	Termination<br>(-H, -OH, or H_2O)	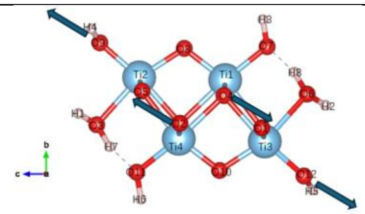<br>Minor vibrations also spread along a axis
380	1DL	Termination<br>(-H, -OH, or H_2O)	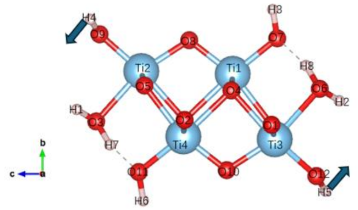<br>Minor vibrations also spread along a axis
361	1DL	Backbone<br>(Ti-O)	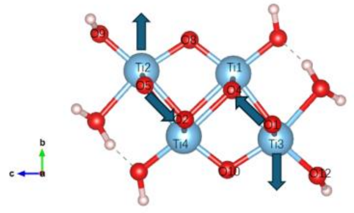

324	1DL	Termination<br>(-H, -OH, or H_2O)	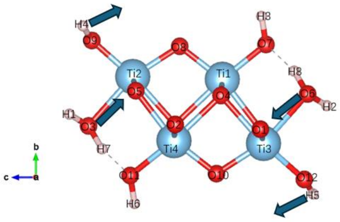
307	1DL	Termination<br>(-H, -OH, or H_2O)	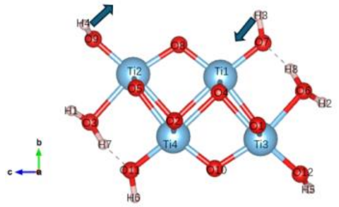<br>Minor vibrations also spread along a axis
286	2D LT	Backbone<br>(Ti-O)	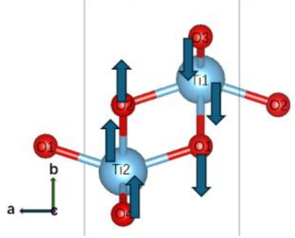
270	1DL	Backbone<br>(Ti-O)	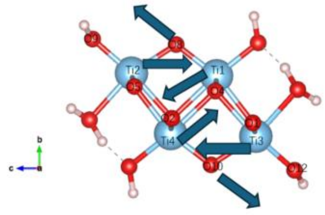<br>Minor vibrations also spread along a axis
215	2D LT	Backbone<br>(Ti-O)	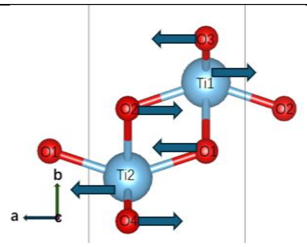

212	1DL	Backbone (Ti-O)	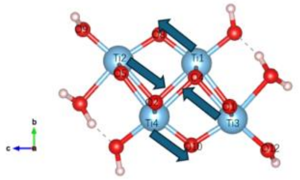 Minor vibrations also spread along a axis
183	1DL	Backbone (Ti-O)	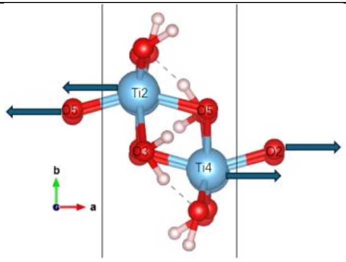 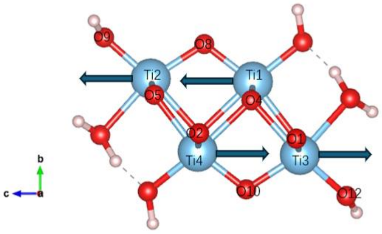
135	1DL	Backbone (Ti-O)	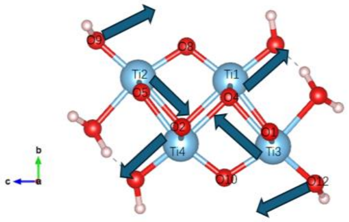 Minor vibrations also spread along

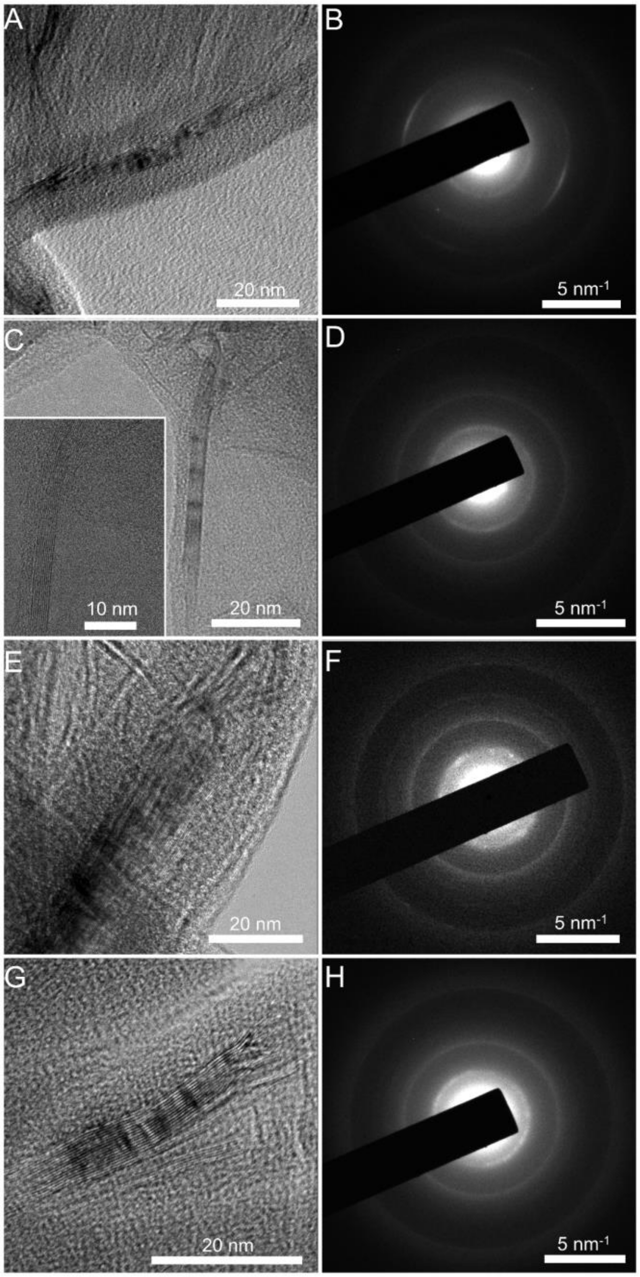

Figure S13. TEM micrographs and associated SAED patterns for various 1DL colloids drop cast onto TEM grids. (A) 10 g/L, (C) 1 g/L, (E) 0.1 g/L, and (G) 0.01 g/L colloids. Inset in (C) shows area of high order in the center of the zoomed-out image. SAED patterns of regions associated with, (B) 10 g/L, (D) 1 g/L, (F) 0.1 g/L, and (H) 0.01 g/L.

Table S2. XRD peak characteristics and Scherrer equation results. Based on 200 peak calculated from data in **Figure 6**.

	200 peak			
[1DL] (g/L)	2Θ (°)	d (Å)	FWHM (°)	Crystallite Size (nm)
10	48.7	1.9	1.16	8.1
1	48.24	1.9	0.92	9.5
0.1	48.72	1.9	1.08	8.1

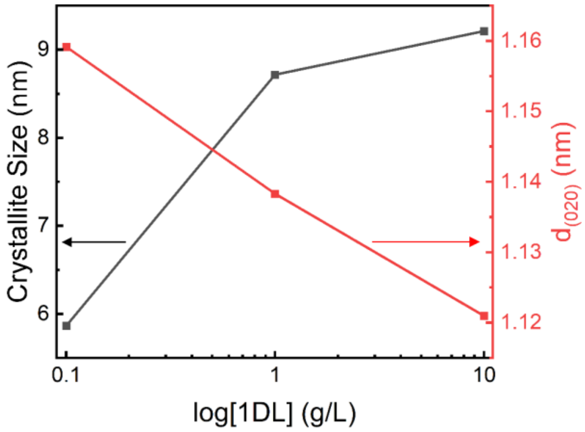

Figure S14. Analysis of 020 XRD peak shown in Figure 6. Semi-log plot of (left axis, black) crystalline sizes in *b*-direction using the Scherrer equation[2] and (right axis, red) *d*-spacing associated with (020) peaks.

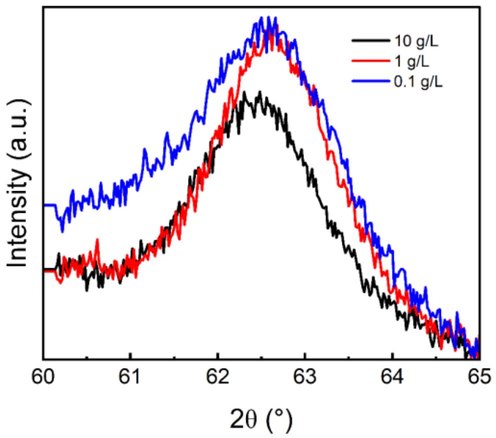

Figure S15. Slow-scan XRD patterns of the high angle (2Θ = 60-65°) region of finely crushed 1DL filtered films. Data utilized for Scherrer equation analysis presented in **Table 1**.

Supplemental References:

(1) García-Tarrés, L.; Guàrdia, E. Hydration and Dynamics of a Tetramethylammonium Ion in Water: A Computer Simulation Study. *The Journal of Physical Chemistry B* **1998**, *102* (38), 7448-7454. DOI: 10.1021/jp981427j.

(2) Patterson, A. L. The Scherrer Formula for X-Ray Particle Size Determination. *Physical Review* **1939**, *56* (10), 978-982. DOI: 10.1103/physrev.56.978.
